# Enhanced Cognitive Effects of Demethoxycurcumin, a Natural Derivative of Curcumin on Scopolamine-Induced Memory Impairment in Mice

**DOI:** 10.3390/molecules21081022

**Published:** 2016-08-05

**Authors:** Dong Wook Lim, Hyun Jung Son, Min Young Um, In-Ho Kim, Daeseok Han, Suengmok Cho, Chang-Ho Lee

**Affiliations:** 1 Research Group of Innovative Special Food, Korea Food Research Institute, Seongnam 13539, Korea; neodw4015@kfri.re.kr (D.W.L.); Son.Hyun-jung@kfri.re.kr (H.J.S.); myum@kfri.re.kr (M.Y.U.); skihs@kfri.re.kr (I.-H.K.); imissu@kfri.re.kr (D.H.); smcho@kfri.re.kr (S.C.); 2 Division of Food Biotechnology, University of Science & Technology, Daejeon 34113, Korea

**Keywords:** demethoxycurcumin, scopolamine, acetyltransferase, passive avoidance task, Morris water maze

## Abstract

In the present study, we examined the ameliorating effects of demethoxycurcumin (DMC) on memory impairment induced by scopolamine using passive avoidance and Morris water maze tests in mice. Moreover, to determine the neurobiological effects underlying the ameliorating effects of the DMC, choline acetyltransferase (ChAT) immunoreactivity was evaluated in mice exposed to scopolamine. Our results demonstrated that chronic oral administration (28 days) of DMC (10 mg/kg) improved scopolamine-induced learning impairment in the passive avoidance task and memory impairment in the Morris water maze. Moreover, Choline acetyltransferase (ChAT) activity in the DMC-treated group was significantly increased to 33.03% compared with the control group. Our present finding suggests that DMC ameliorates memory impairments induced by scopolamine treatment through reversing the reduction of hippocampal ChAT expression in mice.

## 1. Introduction

Curcuminoids are the major active components of Turmeric (rhizome of *Curcuma longa*), one of the earliest known and most important edible crude herbs, which is used in food coloring, for cooking, and in traditional folk medicines [1]. Curcuminoids consist of a mixture of curcumin (75%–80%), demethoxycurcumin (DMC, 15%–20%), and bisdemethoxycurcumin (BDMC, 3%–5%) [2].

Curcumin is present in the largest quantity in curcuminoids, and it is one of the well-studied biologically active molecules of Turmeric, exhibiting antioxidant, anti-inflammatory, and anticancer properties [3]. Over the past half century, previous studies have indicated curcumin’s therapeutic potential against a wide range of human diseases [4]. Owing to its potent anti-inflammatory property, curcumin can be a useful agent in the treatment of neuroinflammation in Alzheimer’s disease (AD), where the production of amyloid-β (Aβ) and the concurrent production reactive oxygen species (ROS) synergistically increases the damage to the neurons [5]. This report was supported by the finding that curcumin decreases Aβ-ROS-related inflammation and Aβ burden in Aβ precursor protein (APP) transgenic mice [6]. Recently, Zhang et al. reported that DMC exhibited a stronger inhibitory activity on nitric oxide (NO) and tumor necrosis factor-α (TNF-α) production compared to curcumin in lipopolysaccharide-activated rat primary microglia [7]. Additionally, more reports have demonstrated the stronger activity of DMC compared to curcumin [8,9,10]. From the above reports, it is hypothesized that the anti-inflammatory action of DMC might have potential enhanced cognitive effects as agents for AD.

In the present study, we examined the ameliorating effects of DMC on memory impairment induced by scopolamine using passive avoidance and Morris water maze tests in mice. Moreover, to determine the neurobiological effects underlying the ameliorating effects of the DMC, choline acetyltransferase (ChAT) immunoreactivity was evaluated in mice exposed to scopolamine.

## 2. Results

### 2.1. Effects of DMC on Scopolamine-Induced Memory Impairment of the Passive Avoidance Response

As shown in Table 1, scopolamine-treated mice showed a significantly decreased latency of reaction (25.2 ± 10.0 s) compared to the vehicle-treated normal group (115.0 ± 36.6 s, *p* < 0.001), indicating that scopolamine impaired the acquisition of the passive avoidance response. The latency times of DMC-treated mice were significantly increased compared to the scopolamine-treated control group (65.9 ± 12.4 s vs. 25.2 ± 10.0 s, *p* < 0.05).

### 2.2. Effects of DMC on Scopolamine-Induced Memory Impairment in Morris Water Maze

**Figure 1:**
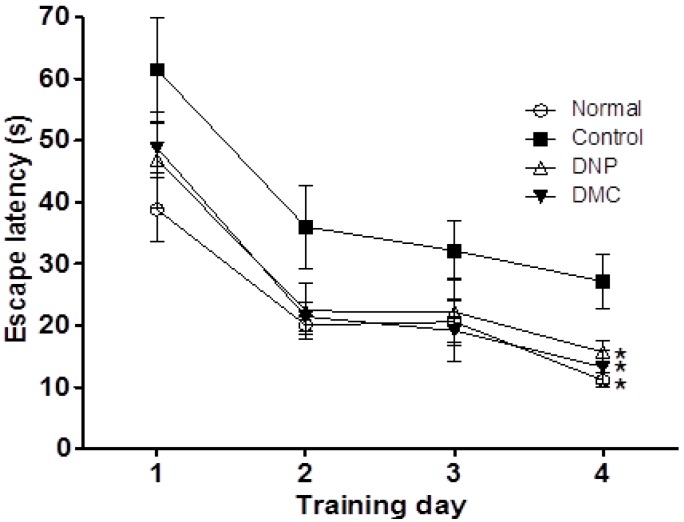
Effect of DMC on hidden-platform acquisition training in Morris water maze. DMC (10 mg/kg, p.o.) and DNP (2 mg/kg, p.o.) were administered once a day for 28 days. Memory impairment was induced by scopolamine (1 mg/kg, i.p.). Comparison of escape latency between DMC-treated groups (*n* = 8 per group) and scopolamine-treated control group during four training days. Data are presented as mean ± SEMs (*n* = 8 per group). * *p* < 0.05 as compared to the scopolamine-treated control group.

**Figure 2:**
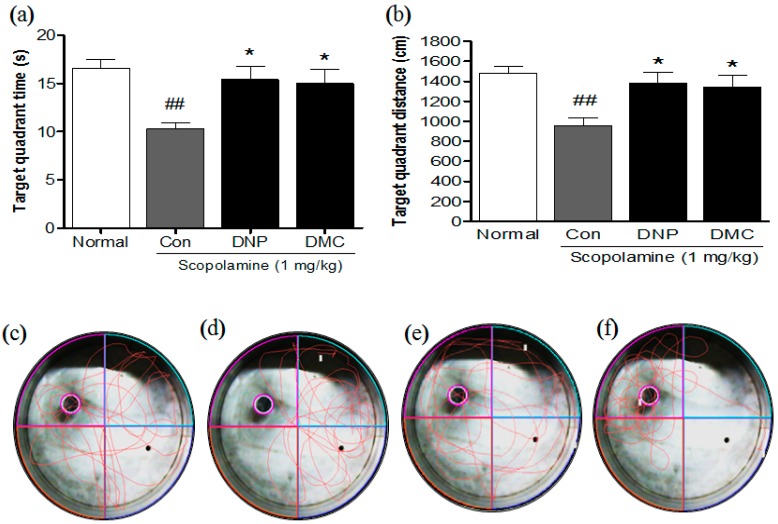
Effect of DMC on scopolamine-induced memory impairment in Morris water maze. DMC (10 mg/kg, p.o.) and DNP (2 mg/kg, p.o.) were administered once a day for 28 days. Memory impairment was induced by scopolamine (1 mg/kg, i.p.). Comparison of the (**a**) swimming time and (**b**) distance for each mouse spent in the target quadrant for probe trial within 120 s. Representation of swimming paths during the water Morris maze in the last day of evaluation (day 5): (**c**) Normal (distilled water); (**d**) scopolamine (1 mg/kg); (**e**) DNP (2 mg/kg); (**f**) DMC (10 mg/kg). Data are presented as mean ± SEMs (*n* = 8 per group). ^##^ *p* < 0.01 as compared to the vehicle-treated normal group; * *p* < 0.05 as compared to the scopolamine-treated control group.

On the fourth day, the probe trial testing was performed by removing the platform and allowing each mouse to swim freely for 120 s inside the pool. The escape latency was averaged for two trial sessions and for each mouse. The vehicle-treated normal group rapidly learned the location of the platform and would swim quickly across the pool to reach it. The scopolamine-treated control group showed significantly delayed escape latency time compared with the control group. However, the escape latency times of DMC-treated mice were significantly increased compared to the control group at the fourth day (Figure 1, 65.9 ± 12.4 s vs. 25.2 ± 10.0 s, *p* < 0.05). In the probe trial following the last training session, DMC at a dose of 10 mg/kg significantly increased the swimming time and distance in the target quadrant after the platform was removed (Figure 2a,b, *p* < 0.05).

### 2.3. Effect of DMC on ChAT Expression in the Hippocampus CA3 Region

**Figure 3:**
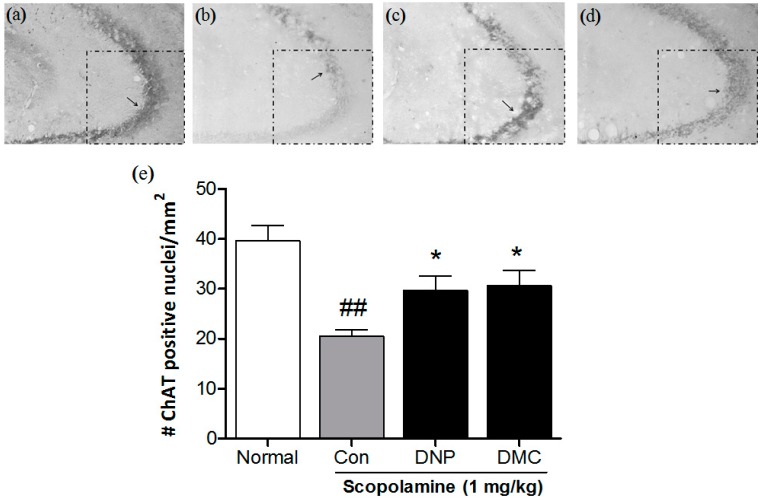
Effects of DMC on the choline acetyltransferase (ChAT) expression in the CA3 region of the hippocampus. Representative photomicrographs show ChAT-positive nuclei in the CA3 of (**a**) Normal (distilled water); (**b**) scopolamine (1 mg/kg); (**c**) DNP (2 mg/kg); and (**d**) DMC (10 mg/kg). Arrows indicate examples of ChAT-positive neurons; (**e**) Columns show the means ± SEMs (*n* = 8) values of ChAT expression in the CA3 region of the hippocampus. ^##^ *p* < 0.01 as compared to the vehicle-treated normal group; * *p* < 0.05 as compared to the scopolamine-treated control group.

To examine whether DMC affected the neural responses in mice exposed to scopolamine, ChAT expression was measured in the hippocampus using immunohistochemistry. Decreased activation of ChAT in the scopolamine-treated control group was observed in the CA3 region of the hippocampus. Importantly, treatment with DMC at a dose of 10 mg/kg significantly inhibited the decrease of ChAT as compared with that in the scopolamine-treated control group (Figure 3).

## 3. Discussion

In the present study, we examined the ameliorating effects of DMC on memory impairment induced by scopolamine using passive avoidance and Morris water maze tests in mice. Our results demonstrated that chronic oral administration (28 days) of DMC (10 mg/kg) significantly improved scopolamine-induced learning impairment in the passive avoidance task and memory impairment in the Morris water maze. Moreover, Choline acetyltransferase (ChAT) activity in the DMC-treated group was significantly increased to 33.03% compared with the control group. The passive avoidance and Morris water maze tests are useful experimental tools for the estimation of standard learning and memory [11,12]. Scopolamine—a muscarinic receptor antagonist—is a standard drug used to induce cognitive deficits in healthy humans [13], and it is widely used to study cognitive deficits in experimental animals [14]. Blockade of central muscarinic receptors may induce a pattern of cognitive impairment in Alzheimer’s disease (AD) patients [15]. Therefore, donepezil (DNP)—an acetylcholinesterase (AChE) inhibitor, and the most widely-used treatment for AD—was used as a positive control [16,17]. Here, chronic administration of DMC inhibited reductions in step-through latency induced by scopolamine. In our Morris water maze tests, the vehicle-treated normal group exhibited rapid reductions in escape latency time to find the location of the platform from day two, and reached a stable escape latency time during the training trials. However, the scopolamine-treated control group did not reduce the escape latency time from days two to four, indicating memory impairment induced by scopolamine. At the probe trial session on day four, chronic administration of DMC was significantly shortened escape latency time. In the probe trial following the last training session, DMC administration improved the swimming time and distance within the target quadrant compared to the scopolamine-treated control group. Consistent with our findings from the DNP-treated group, these results suggest that DMC ameliorates memory impairments induced by scopolamine treatment through rescue of the acetylcholine system.

The ameliorating effect of DMC was confirmed by quantitative analysis of Choline acetyltransferase (ChAT) immunoreactivity. Cognitive functions are strongly dependent on central cholinergic neurotransmission [18], and the cholinergic nerve system plays a major role in mediating cognitive performances [19]. ChAT—the enzyme responsible for acetylcholine (Ach) biosynthesis—is presently the most specific marker for monitoring the functional state of cholinergic neurons in the peripheral and central cholinergic nervous system [20]. ChAT activity has been found to be significantly reduced not only in AD patients [21], but also in Parkinson’s disease (PD) patients [22]. It is also reported that the degree of the reduction in cerebral ChAT activity is significantly correlated with the severity of dementia [23]. Therefore, it is generally accepted that the cellular loss and dysfunction of cholinergic neurons results in the development of dementia in AD and other types of brain disease [20]. Consistent with these reports, we found that chronic treatment with DMC at a dose of 10 mg/kg inhibited the decrease of ChAT-positive nuclei in the hippocampus CA3 region. Although we did not determine ChAT phosphorylation and ChAT-dependent transactivation in hypothalamic CA3, our findings suggest that DMC reverses the downregulation of hippocampal ChAT expression in mice exposed to the scopolamine.

## 4. Materials and Methods

### 4.1. Animals

Male ICR mice (6 weeks old; NARA biotech, Seoul, Korea) weighing 30–35 g were housed at four mice per cage under a controlled temperature (21 ± 2 °C) and a 12-h light/dark cycle (lights on at 7:00 and lights off at 19:00). The mice were allowed at least 1 week for acclimatization before the experiments. All animal protocols were approved by the Institutional Animal Care and Use Committee (IACUC) of the Korea Food Research Institute.

### 4.2. Drugs and Treatment

Scopolamine hydrobromide, demethoxycurcumin (DMC), and donepezil hydrochloride monohydrate (DNP) were purchased from Sigma (St. Louis, MO, USA). DMC (10 mg/kg) or DNP (2 mg/kg) as a positive control were dissolved in corn oil and orally administered once a day for 28 consecutive days. The normal group was administered the vehicle solution (1 mL/kg, p.o.) using the same schedule of administration. On day 28, the mice were exposed to the behavioral experiments 1 h after the administration.

### 4.3. Passive Avoidance Test

The passive avoidance test was carried out as previously described [24]. A modified passive avoidance test was used to assess the effect of DMC on scopolamine-induced memory impairment. The apparatus for the step-through passive avoidance test was an automated shuttle-box (Gemini Avoidance System, San Diego, CA, USA), divided into a lighted compartment and a dark compartment of the same size (20 cm × 20 cm × 20 cm) by a wall with a guillotine door (5 cm × 5 cm). Briefly, in the training trial, a mouse was placed in the lighted chamber, and when the mouse entered the dark chamber, a 0.3 mA electrical shock of 3 s duration was delivered through floor grids. DMC (10 mg/kg, p.o.) or donepezil (2 mg/kg, p.o.) as a positive control were given orally 1 h before treatment with scopolamine. Memory impairment was induced by treatment with scopolamine (1 mg/kg, i.p.), and the passive avoidance test was performed 30 min after treatment with scopolamine. A retention trial was performed 24 h after the training trial, and latency time to enter the dark chamber were measured for 300 s.

### 4.4. Morris Water Maze Test

The Morris water maze test was carried out as previously described [25]. Briefly, the circular pool was filled to a depth height of 30 cm with water at room temperature (22 ± 1 °C) and was made opaque by the addition of 500 mL India ink. The four quadrants of the pool were designed as starting positions: east (E), south (S), west (W), and north (N). An escape platform (diameter 6.5 cm) was set 1 cm below the surface of the water and placed in a constant position in the middle of the NW quadrant. The mice were given two trial sessions each day for four consecutive days, and the escape latencies were recorded. Once the mouse located the platform, it was permitted to remain on it for 30 s. If the mice failed to find the platform within 120 s, they were placed on the platform for 30 s and then removed from the pool. The escape latency and swim path length were recorded by the video-tracking system (SMART, Panlap, Barcelona, Spain).

### 4.5. Choline Acetyltransferase Immunohistochemistry

Mice were sacrificed following the passive avoidance test, and their brains were fixed through the ascending aorta with 0.9% saline, followed by 100 mL of cold 0.1 M phosphate buffer (PB) containing 4% paraformaldehyde (PFA). The fixed brains were cut into 30 µm sections on a cryostat (CM1850; Leica, Heidelberg, Germany). Immunohistochemistry staining was performed on 30 µm sections using polyclonal antibodies specific for ChAT (1:2000 dilution; AB 1582, Chemicon, CA, USA) followed by exposure to a biotinylated anti-sheep antibody (1:500 dilution, BA 1000, Vector Labs, Burlington, ON, Canada). The sections were reacted with an avidin–biotin–peroxidase complex (1:50 dilution, Elite ABC kit, Vector Laboratories) at room temperature for 60 min, and the avidin–biotin complex was visualized with 0.05% 3,3-diaminobenzidine (DAB, Sigma) and 0.02% H_2_O_2_. Images of immunohistochemically stained sections were captured by a camera mounted on an Olympus BX-51 microscope (Olympus Optical, Tokyo, Japan).

### 4.6. Statistical Analysis

Data analysis was performed using one-way analysis of variance (ANOVA) followed by Tukey’s *post hoc* test using Prism 5 (GraphPad Software, Inc., San Diego, CA, USA) for multigroup comparisons. Results of *p* < 0.05 were considered statistically significant. All results are expressed as mean ± standard error of the mean (SEMs).

## 5. Conclusions

In conclusion, the present study demonstrated that chronic oral administration of DMC significantly reversed scopolamine-induced memory impairments in mice as evaluated by the passive avoidance test, and also improved escape latency in the Morris water maze, as indicated by inhibition of the reduction of ChAT expression in the hippocampal CA3 region. Our present findings suggest that DMC (or a constituent of DMC) has preventive or therapeutic potential for the treatment of memory impairment-related diseases such as AD.

## Figures and Tables

**Table 1:** Effect of demethoxycurcumin (DMC) on scopolamine-induced memory impairment in the passive avoidance test.

Group	Step-Through Latency (s)
Normal (vehicle-treated normal group)	115.0 ± 36.6
Scopolamine + vehicle	25.2 ± 10.0 ^a^
Scopolamine + DNP 2 mg/kg	62.7 ± 36.0 ^b^
Scopolamine + DMC 10 mg/kg	65.9 ± 12.4 ^b^

DMC (10 mg/kg, p.o.) and DNP (donepezil) (2 mg/kg, p.o.) were administrated once a day for 28 days. Memory impairment was induced by treatment with scopolamine (1 mg/kg, i.p.), and the passive avoidance test was performed 30 min after treatment with scopolamine. Data are presented as mean ± SEMs (*n* = 8 per group). ^a^ *p* < 0.001 as compared to the vehicle-treated normal group; ^b^ *p* < 0.05 as compared to the scopolamine-treated control group.

## Abbreviations

The following abbreviations are used in this manuscript:

AChE	Acetylcholinesterase
AD	Alzheimer’s disease
APP	Aβ precursor protein
BDMC	Bisdemethoxycurcumin
ChAT	Choline acetyltransferase
DMC	Demethoxycurcumin
DNP	Donepezil
PD	Parkinson’s disease
ROS	Reactive oxygen species
TNF-α	Tumor necrosis factor-α

## References

[1] Goel A., Kunnumakkara A.B., Aggarwal B.B. (2008). Curcumin as “curecumin”: From kitchen to clinic. Biochem. Pharmacol..

[2] Hung C.M., Su Y.H., Lin H.Y., Lin J.N., Liu L.C., Ho C.T., Way T.D. (2012). Demethoxycurcumin modulates prostate cancer cell proliferation via AMPK-induced down-regulation of HSP70 and EGFR. J. Agric. Food Chem..

[3] Aggarwal B.B., Harikumar K.B. (2009). Potential therapeutic effects of curcumin, the anti-inflammatory agent, against neurodegenerative, cardiovascular, pulmonary, metabolic, autoimmune and neoplastic diseases. Int. J. Biochem. Cell Biol..

[4] Gupta S.C., Patchva S., Aggarwal B.B. (2013). Therapeutic roles of curcumin: Lessons learned from clinical trials. AAPS J..

[5] Ray B., Lahiri D.K. (2009). Neuroinflammation in alzheimer’s disease: Different molecular targets and potential therapeutic agents including curcumin. Curr. Opin. Pharmacol..

[6] Lim G.P., Chu T., Yang F.S., Beech W., Frautschy S.A., Cole G.M. (2001). The curry spice curcumin reduces oxidative damage and amyloid pathology in an alzheimer transgenic mouse. J. Neurosci..

[7] Zhang L., Wu C., Zhao S., Yuan D., Lian G., Wang X., Wang L., Yang J. (2010). Demethoxycurcumin, a natural derivative of curcumin attenuates LPS-induced pro-inflammatory responses through down-regulation of intracellular ROS-related mapk/nf-kappab signaling pathways in N9 microglia induced by lipopolysaccharide. Int. Immunopharmacol..

[8] Luthra P.M., Kumar R., Prakash A. (2009). Demethoxycurcumin induces Bcl-2 mediated G2/M arrest and apoptosis in human glioma U87 cells. Biochem. Biophys. Res. Commun..

[9] Yodkeeree S., Chaiwangyen W., Garbisa S., Limtrakul P. (2009). Curcumin, demethoxycurcumin and bisdemethoxycurcumin differentially inhibit cancer cell invasion through the down-regulation of mmps and upa. J. Nutr. Biochem..

[10] Ahmed T., Gilani A.H. (2009). Inhibitory effect of curcuminoids on acetylcholinesterase activity and attenuation of scopolamine-induced amnesia may explain medicinal use of turmeric in alzheimer’s disease. Pharmacol. Biochem. Behav..

[11] Bejar C., Wang R.H., Weinstock M. (1999). Effect of rivastigmine on scopolamine-induced memory impairment in rats. Eur. J. Pharmacol..

[12] Fibiger H.C. (1991). Cholinergic mechanisms in learning, memory and dementia: A review of recent evidence. Trends Neurosci..

[13] Klinkenberg I., Blokland A. (2010). The validity of scopolamine as a pharmacological model for cognitive impairment: A review of animal behavioral studies. Neurosci. Biobehav. Rev..

[14] Jeong E.J., Lee K.Y., Kim S.H., Sung S.H., Kim Y.C. (2008). Cognitive-enhancing and antioxidant activities of iridoid glycosides from scrophularia buergeriana in scopolamine-treated mice. Eur. J. Pharmacol..

[15] Buccafusco J.J., Buccafusco J.J. (2009). The revival of scopolamine reversal for the assessment of cognition-enhancing drugs. Methods of Behavior Analysis in Neuroscience.

[16] Long J.M., Lahiri D.K. (2011). Current drug targets for modulating alzheimer’s amyloid precursor protein: Role of specific micro-rna species. Curr. Med. Chem..

[17] Prasher V.P., Huxley A., Haque M.S. (2002). Down Syndrome Ageing Study, G. A 24-week, double-blind, placebo-controlled trial of donepezil in patients with down syndRome and alzheimer’s disease—Pilot study. Int. J. Geriatr. Psychiatry.

[18] Sarter M., Parikh V. (2005). Choline transporters, cholinergic transmission and cognition. Nat. Rev. Neurosci..

[19] Hasselmo M.E., Sarter M. (2011). Modes and models of forebrain cholinergic neuromodulation of cognition. Neuropsychopharmacology.

[20] Oda Y. (1999). Choline acetyltransferase: The structure, distribution and pathologic changes in the central nervous system. Pathol. Int..

[21] Ikonomovic M.D., Mufson E.J., Wuu J., Bennett D.A., DeKosky S.T. (2005). Reduction of choline acetyltransferase activity in primary visual cortex in mild to moderate alzheimer's disease. Arch. Neurol..

[22] Mattila P.M., Roytta M., Lonnberg P., Marjamaki P., Helenius H., Rinne J.O. (2001). Choline acetyltransferase activity and striatal dopamine receptors in parkinson’s disease in relation to cognitive impairment. Acta Neuropathol..

[23] Sharp S.I., Francis P.T., Elliott M.S., Kalaria R.N., Bajic N., Hortobagyi T., Ballard C.G. (2009). Choline acetyltransferase activity in vascular dementia and stroke. Dement. Geriatr. Cogn. Disord..

[24] Yamada K., Tanaka T., Han D., Senzaki K., Kameyama T., Nabeshima T. (1999). Protective effects of idebenone and alpha-tocopherol on beta-amyloid-(1-42)-induced learning and memory deficits in rats: Implication of oxidative stress in beta-amyloid-induced neurotoxicity in vivo. Eur. J. Neurosci..

[25] Brandeis R., Brandys Y., Yehuda S. (1989). The use of the morris water maze in the study of memory and learning. Int. J. Neurosci..

